# Use of hand hygiene agents as a surrogate marker of compliance in hungarian long-term care facilities: first nationwide survey

**DOI:** 10.1186/2047-2994-4-S1-P294

**Published:** 2015-06-16

**Authors:** R Szabó, J Morvai, F Bellissimo-Rodrigues, D Pittet

**Affiliations:** 1 Department of Hospital Epidemiology and Hygiene, National Center for Epidemiology, Budapest, Hungary; 2 School of PH.D. Studies, Semmelweis University, Budapest, Hungary; 3 Infection Control Programme and World Health Organization (WHO) Collaborating Centre on Patient Safety, University of Geneva Hospitals and Faculty of Medicine, Geneva, Switzerland

## Introduction

Hand hygiene practice is an important measure for preventing infections in long-term care facilities (LTCFs). However, low compliance with hand hygiene has been reported in a number of studies.

## Objectives

The purpose of this study was to provide an overview of the first reference data collected on alcohol-based handrub (ABHR) and antiseptic soap consumption, as surrogate markers for hand hygiene compliance by healthcare workers (HCWs) in Hungarian LTCFs. The objective was to inform stakeholders on the need of hand hygiene improvement in these settings.

## Methods

Between 5 May and 30 September 2014, we conducted a nationwide, cross-sectional survey using a standardized self-administered questionnaire; all Hungarian LTCFs were eligible. The Statistical Package for Social Sciences (SPSS) version 20.0 was used for data analysis.

## Results

The questionnaire was completed by 354 LTCFs, representing 24% of all Hungarian LTCFs. In total, the median consumption of ABHR and antimicrobial soap was 15.5 L (IQR, 0-800 L) and 60 L (IQR, 0-1,680 L) per LTCFs annually, and 2.2 mL (IQR, 0.4-9.1 mL) and 12.1 mL (IQR, 0.7-32.8 mL) per HCWs per day in 2013, respectively. The estimated number of hand hygiene actions was 0.6 hygienic handrub/HCW per day (IQR, 0-12.8/HCWs) and 2.4 hygienic handwashing/HCW per day (IQR, 0-21.9/HCWs; *P*=.001), respectively.

## Conclusion

This study suggests that non-compliance with hand hygiene is a significant problem in Hungarian LTCFs. Based on our results, there is an urgent need for a nationwide multimodal hand hygiene promotion strategy including education and performance monitoring and feedback in all LTCFs. Furthermore, monitoring of ABHR consumption constitute an additional component of the existing National Nosocomial Surveillance system.

## Disclosure of interest

None declared.

